# Generative AI for spatial tumor growth on MRI: a proof-of-principle study in pediatric diffuse midline glioma

**DOI:** 10.1186/s12916-026-04911-y

**Published:** 2026-05-18

**Authors:** Daria Laslo, Julia Wolleb, Maria Monzon, Raimund Kottke, Selma Sirin, Timothy Müller, Dror Suhami, Franziska Vogt, Aashim Bhatia, Deep B. Gandhi, Anahita Fathi Kazerooni, Ariana M. Familiar, Thien Nguyen, Zhifan Jiang, Abhijeet Parida, Nicolas U. Gerber, Ana Guerreiro Stücklin, Ali Nabavizadeh, Javad Nazarian, Marius George Linguraru, Andreas M. Rauschecker, Sabine Müller, Catherine Jutzeler, Sarah Brüningk

**Affiliations:** 1 D-HEST, ETH Zurich, Zurich, Switzerland; 2 Swiss Institute of Bioinformatics, Lausanne, Switzerland; 3 Department of Biomedical Informatics and Data Science, Yale School of Medicine, New Haven, CT USA; 4 Department of Diagnostic Imaging, University Children’s Hospital Zurich, Zurich, Switzerland; 5 Neurosurgery, University Children’s Hospital Zurich, Zurich, Switzerland; 6 Center for Intelligent Imaging, Department of Radiology & Biomedical Imaging, University of California San Francisco, San Francisco, USA; 7 The Center for Data Driven Discovery of Biomedicine, The Children’s Hospital of Philadelphia, Philadelphia, USA; 8 Division of Pediatric Oncology, Stanford University, Stanford, USA; 9 Sheikh Zayed Institute, Children’s National Hospital, Washington, DC USA; 10 ETSIT, Universidad Politécnica de Madrid, Madrid, Spain; 11 Department of Pediatrics, University Children’s Hospital Zurich, Zurich, Switzerland; 12 University of Pennsylvania, Philadelphia, USA; 13 Center for Cancer and Immunology Research, Children’s National Hospital, Washington, DC USA; 14 School of Medicine and Health Sciences, George Washington University, Washington, DC USA; 15 Neurosurgery and Pediatrics, University of California San Francisco, San Francisco, USA; 16 Department of Radiation Oncology, Inselspital, Bern University Hospital and University of Bern, Bern, Switzerland; 17 Department of Digital Medicine, University of Bern, Bern, Switzerland

**Keywords:** Generative AI, Denoising Diffusion Models, DMG, Brain tumor, Anatomical tumor growth

## Abstract

**Background:**

Magnetic resonance imaging (MRI) is a cornerstone of non-invasive diagnosis and response monitoring in neuro-oncology, and predictions of spatial tumor progression conditioned on the patients’ anatomy are increasingly important. We present a proof-of-principle of personalized spatial tumor progression on MRI through generative AI, focusing on pediatric Diffuse Midline Glioma (DMG).

**Methods:**

We employed guided Denoising Diffusion Implicit Models (DDIM) to model anatomical tumor growth in pediatric DMGs on MRI. Multiparametric scans from adult (*n* = 1,251) and pediatric (*n* = 144) patients from the BraTS23 challenge were used to train a slice-based framework, conditioned on baseline scans and a target tumor size. Repeated image generations produce probabilistic tumor growth maps highlighting likely regions of progression. The realism of the generated MRIs was evaluated quantitatively and qualitatively through expert assessment. Spatial growth predictions were validated against an independent dataset of longitudinal MRI scans from a multi-institutional pre-radiotherapy DMG dataset (*n* = 178 paired slices).

**Results:**

We generated anatomically coherent, patient-specific T2-FLAIR (fluid-attenuated inversion recovery) MRI axial slices. Quantitative measures and expert evaluations confirmed the high quality of the generated images, which trained radiologists were unable to reliably distinguish from real scans (accuracy 0.53 ± 0.03). While radiomic features analyses showed good agreement (83% non-significant features) between synthetic and real images, a classifier detected subtle pixel-wise differences (accuracy of 0.69). Tumor growth probability maps aligned well with true tumor growth observed in follow-up imaging, obtaining a mean continuous DICE score of 0.79 ± 0.13.

**Conclusions:**

We present guided DDIMs as a predictive tool for spatial tumor growth, illustrated for the progression of DMGs, that demonstrates potential for its integration in personalized radiotherapy planning. Our comprehensive image quality analysis highlights the importance of carefully evaluating synthetic data and its integration in research and clinical workflows.

**Supplementary Information:**

The online version contains supplementary material available at 10.1186/s12916-026-04911-y.

## Background

Personalized predictions of tumor growth patterns, that reflect the patient-specific anatomy and tumor burden, are increasingly recognized as a critical component in the field of precision oncology [1]. Medical imaging plays a central role in this process, enabling non-invasive monitoring of tumor progression as well as guiding clinical localized therapy decisions. Magnetic resonance imaging (MRI) is a cornerstone of neuro-oncology for both diagnosis and treatment response assessment, making it a natural foundation of tumor growth predictions. In addition to providing detailed anatomical information, MRI captures important microenvironmental factors reflecting radiosensitivity, including tumor cell density, tumor perfusion, and necrosis [2]. Two independent lines of research, data-driven and mechanism-driven models have previously been applied for image-based tumor growth predictions in the context of adult high grade glioma and glioblastoma (GBM) [3–6]. Recently diffusion models have been adapted for longitudinal modeling of glioma, through TaDiff-Net [7]. Here, we focus on adopting this generative paradigm to pediatric gliomas for modeling pre-radiotherapy (pre-RT) growth in a limited data scenario.

Despite advances in pediatric cancer care, malignant neoplasms are among the leading causes of death in children and adolescents [8]. Particularly concerning are malignant tumors of the central nervous system amongst which diffuse midline glioma (DMG) stands out with a particularly poor prognosis (overall survival < two years) [9]. DMGs are defined by a lysine substitution at position 27 on the H3 histone tail protein (H3K27M) and clinically present as diffuse, infiltrating lesions located within sensitive brain regions (pons, brainstem, and thalamus) [10]. The standard of care for DMG is neoadjuvant radiation therapy with serial radiographic monitoring, and subsequent enrollment in a clinical trial. Standardized criteria such as RAPNO (response assessment in pediatric neuro-oncology) are employed to assess disease progression, relying on hard thresholds (25% increase/decrease for progression/remission) imposed on changes in bidimensional measurements on tumor MRI appearance [11]. Gross tumor resection is largely excluded due to the tumors’ sensitive location and diffuse phenotype. Given frequent imaging and lack of surgical interventions, DMGs are a particularly suitable candidate for proof-of-principle of anatomical tumor growth predictions.

Recently, Denoising Diffusion Implicit Models (DDIMs) [12], as a branch of Denoising Diffusion Probabilistic Models (DDPMs) [13], have been suggested as a new emerging field in the realm of generative computer vision models. DDPMs build on a diffusion process to generate images by learning the data distribution through the reversal of the noising process. Denoising Diffusion Implicit Models (DDIMs) slightly alter the sampling procedure of DDPMs, following a deterministic process. Despite promising first results for adult GBM growth predictions, no validated longitudinal predictions in the context of disease progression have been presented to date, and a focus for DDIM has been on sequence generation and image synthesis [14–18]. While shown to outperform most generative models to date for both natural and medical images, truly quantifying image realism, particularly within the medical domain, remains difficult. Moreover, the downstream impact of integrating synthetic images in newly developed training frameworks is poorly understood.

**Fig. 1:**
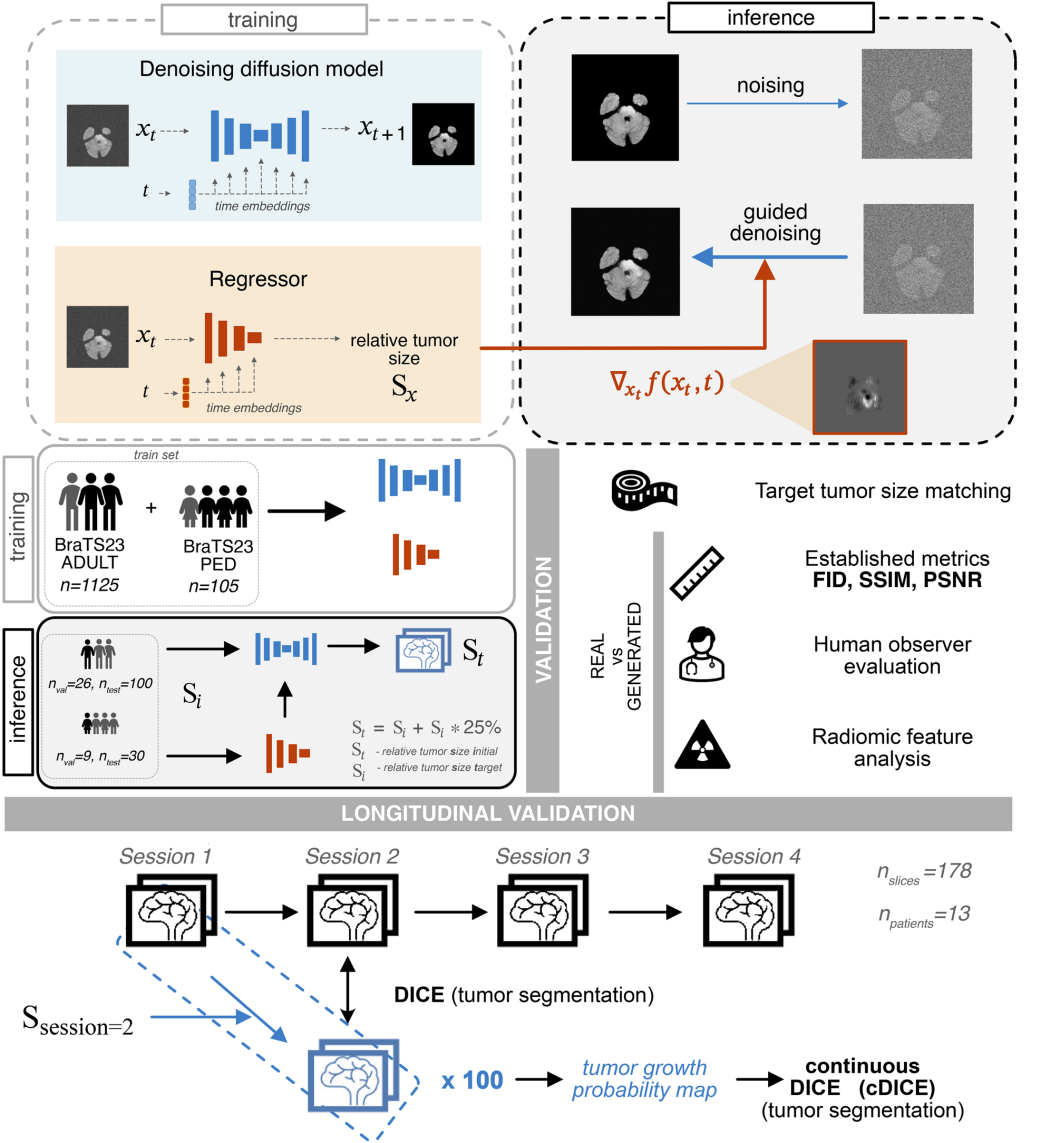
Study overview. A detailed overview of the model development and inference approach (top) along the different methods employed for the validation of the framework, including image quality assessments and longitudinal evaluation (bottom). *BraTS23 ADULT*,* BraTS PED: brain tumor segmentation challenge datasets* [20, 21]; *FID: Frechet inception distance; SSIM: structural similarity index measure; PSNR: peak signal to noise ratio; DICE: Sorensen-Dice coefficient*

Our study offers proof-of-principle for harnessing guided DDIM to conditionally model the spatial patterns of tumor growth, as a first step towards end-to-end longitudinal predictions. The clinical particularities of DMGs offer a unique opportunity to explore the potential of guided DDIMs as a predictive tool. However, capturing the true distribution of a pediatric dataset in light of limited data remains challenging. As outlined in Fig. 1, we aimed to leverage shared anatomical structures between the adult and pediatric brain to generalize across these populations by jointly training on the relevant data subsets. We quantified the realism of the generated images analytically and based on human expert raters. Additionally, radiomic features were extracted to characterize changes occurring in generated images [19]. A comparative analysis of metrics assessing realism was conducted to disentangle distinct aspects of image realism and elucidate potential pitfalls in conventional quality assessments.

## Methods

### Data preparation

Two multi-parametric (T1-weighted (T1), T1-contrast enhanced (T1-CE), T2-weighted (T2), T2-fluid-attenuated inversion recovery (T2-FLAIR)), single-timepoint datasets are included for the model development: BraTS2023 adult GBM images (*n* = 1,251, BraTS-ADULT) and CBTN-CONNECT-DIPGR-ASNR-MICCAI BraTS2023 comprising pediatric DMG and astrocytoma patients (*n* = 144, BraTS-PED) [20, 21]. These are processed multi-center scans, registered to the same anatomical template, and resampled to 1 mm^3^. Based on the tumor segmentation, we retained all relevant axial image slices that are located in tumor-prevalent regions, removing the first inferior slices (commonly empty) and the superior hemisphere brain slices. A minimum tumor area threshold was imposed based on the distribution of tumor areas in the training data determined by the elbow method (adult 16 mm2, pediatric 9 mm2). This excluded slices with smaller or no tumor content, particularly relevant for the training of the tumor size prediction task.

For longitudinal evaluation we retrospectively analyzed a set of 178 pairs of longitudinal pre-RT MRIs from the DMG center Zurich. Ethical approval for this study was granted based on 2022 − 00312 by the North-West and Central Swiss Ethics Commission, permitting retrospective analysis of clinical imaging data. The Cancer Phenomics Imaging Toolkit (CaPTK) was applied to the external dataset, including resampling to 1 mm^3^, SRI atlas registration, and skull-stripping yielding brain masks [22]. Longitudinal images were coregistered based on alignment of the brain masks using the *Dipy* python package. We rescaled the brain volume intensity to [0,1] by min-max normalization for all datasets after applying percentile bounds (0.5, 99.5). A detailed overview of the data is provided in Additional file 1: Table S1 and Additional file 1: Figure S1. Summary statistics for the longitudinal dataset are provided in Additional file 1: Table S2.

### Computational approach

We employed a guided DDIM for the generation of axial 2D MRI slices showing an enlarged tumor relative to a source image [23]. DDPMs iteratively add noise to data and learn the reverse diffusion process to recover the original input. In guided diffusion models, explicit information is incorporated upon denoising, guiding the generation towards a desired result, here, a larger tumor. Our DDPM was trained on the joint training sets from BraTS23-Adult and BraTS23-PED using an Adam optimizer and hybrid loss introduced by Nichol and Dhariwal [24]. The data was split at patient level into training and validation (90% adult, 80% pediatric) and test subsets (10% adult, 20% pediatric) (Additional file 1: Table S1). There was no subject overlap between the training, validation and testing samples. Training was stopped once the validation error reduction between saving iterations (every 5,000 steps) dropped below 5% (160,000 steps). A regression model predicting relative tumor size was independently trained on the same splits, harnessing tumor segmentations. It follows the architecture of the encoder of the diffusion model’s U-Net-like network [25], including time-step embeddings and a final linear activation function and was trained using the mean squared error (MSE) loss and the Adam optimizer for 50,000 steps (until error reduction below 5% in consecutive iterations).

Following Wolleb et al. [17], upon inference, sampling was carried out on a noised version of the given input slice, incorporating regressor guidance at every step using the implicit framework (DDIM) of our trained DDPM. Notably, inference requires specifying an anticipated relative tumor area change, which serves as the reference for the magnitude of regressor guidance. Conditional image generation is governed by two hyperparameters: (i) The number of inference steps, given by the noise level (*NL*), which impacts the preservation of the sample’s original anatomical features. (ii) The strength of the guidance controlled by the regressor scale (*RS*). Additionally, the term referring to the gradient of the regressor is individually scaled for each iteration and image by a dynamic factor that penalizes the deviation from the anticipated change in relative tumor area (Additional file 2: Sects. *Model Architecture and Training* and *Hyperparameter Tuning*).

We identified optimal *RS* (range 100k/**200k**/500k) and *NL* (range 300/**400**/500) in a grid search based on overall image quality metrics (Additional file 1: Figure S2) and the error of the observed vs. target tumor area (25% larger). The same RS and NL values were then applied uniformly to all cases. Tumor segmentations of the generated images were obtained with a vanilla U-Net model (Additional file 2: Sect. *U-Net Segmentation Model*) [25].

### Tumor growth probability maps

The network output depends on the forward diffusion pass, through random noise sampling. By maintaining identical inputs (2D MRI slice, target tumor size) and changing random seed for noise sampling (*n* = 100) an image library is generated. We aggregated this library into a single tumor growth probability map where each pixel is associated with a probability of being a tumor pixel in the newly generated image. Otsu thresholding creates tumor growth area segmentations. Only tumor growth in the periphery of the original tumor (a dilation enabling tripling of the tumor) was considered to minimize the impact of out-of-field generations. Resulting axial growth maps (*n* = 10) were stacked in 3D to inspect the volumetric translation of our generated 2D slices.

### Image generation quality quantification

#### Metrics

We used the Frechet Inception Distance (FID), the structural similarity index measure (SSIM), and peak signal to noise ratio (PSNR) to quantify the quality of the generated vs. real MRIs. FID assesses the distance between the distribution of the real samples and that of generated ones, making it prone to dataset size bias [26]. SSIM considers the inter-dependencies between neighboring pixels, including luminance and contrast to approximate structural similarity, while PSNR assesses the ratio between the signal and the distorting noise, a higher value indicating a better image quality. Only slices with tumor annotations were included in this computation.

#### Radiomic features

Radiomic features refer to pixel value-based and shape features extracted from medical images, including simple (e.g., mean, standard deviation) and complex (e.g., grey level non-uniformity) statistics. Here, we compare radiomic features from generated and real MRI scans to assess the realism and quality of the generated images beyond image similarity metrics. Radiomic features (*n* = 1,032), including all those after applying all standard filters (e.g., Laplacian of Gaussian, Wavelet), were extracted using the pyradiomics package [27] from all 2D slices containing tumor. We excluded constant and highly correlated features (Pearson’s coefficient ≥ 0.95) before testing distribution-level differences by Kolmogorov-Smirnov test (multiple testing was accounted for by Bonferroni method, alpha = 0.05). To ensure a robust evaluation, additional tests were carried out between the train and test set as well as between the test set and its version run through the DDIM without any changes to the tumor size (‘diffused’).

#### Human observers

Five pediatric neuroradiology domain experts (three board-certified neuroradiologists and pediatric radiologists, two neuroradiology fellows) classified 200 2D T2-FLAIR scans (100 real test set slices and corresponding generated images, resulting in a perfectly balanced dataset) - reviewers were blinded to the fraction of real/generated images and the fact that generated images were 25% enlarged. Images were provided as axial 2D slices in a PNG format. The classification was done at the expert’s convenience in one sitting. Additionally, a U-Net based classifier (Additional file 2: Sect.  *U-Net Classifier*) was trained to perform the same task, and performance was compared to that of the human observers via accuracy, precision, recall and specificity.

### Longitudinal external evaluation

All pre-RT tumor-containing slices showing a 10–100% tumor area increase for the external DMG cohort were considered for longitudinal evaluation, resulting in the selection of 178 pairs of consecutive 2D MRI slices from 13 patients. All image pairs were acquired prior to radiotherapy, the standard of care for DMGs. Concurrent chemotherapy may have been administered but precise timing relative to imaging was unavailable. Images were segmented and reviewed by trained personnel under the guidance of a board-certified neuroradiologist [28]. DDIMs were guided to generate enlarged tumor images for the consecutive time point based on the known target size. The resulting tumor growth probability maps (*n* = 100 inferences) were thresholded (Otsu) and the predicted whole tumor area was compared to the true follow-up image by continuous DICE (cDICE) score [29]. To further assess spatial correspondence, we computed the 95th percentile Hausdorff distance (HD95) between the generated and follow-up images, as well as between the baseline and follow-up scans. The difference in HD95 $({\Delta} HD\mathrm{95}={\mathrm{HD95}}_{\mathrm{baseline}\to \mathrm{target}}-{\mathrm{HD95}}_{\mathrm{generated}\to \mathrm{target}})$ was used to quantify the improvement in spatial proximity.

### Error analysis

To identify failure modes in the growth generation, we computed Spearman’s rank correlation ($\:\rho\:$) between predictors of model performance and downstream cDICE computed between the segmentations of the generated image and the ground truth follow-up MRI scan. Specifically, we examined the absolute error in the regressor-predicted baseline tumor size, as well as the specific growth rate. Segmentations of generated images were obtained through the thresholding of difference maps (generated - baseline).

## Results

### Model development

**Fig. 2:**
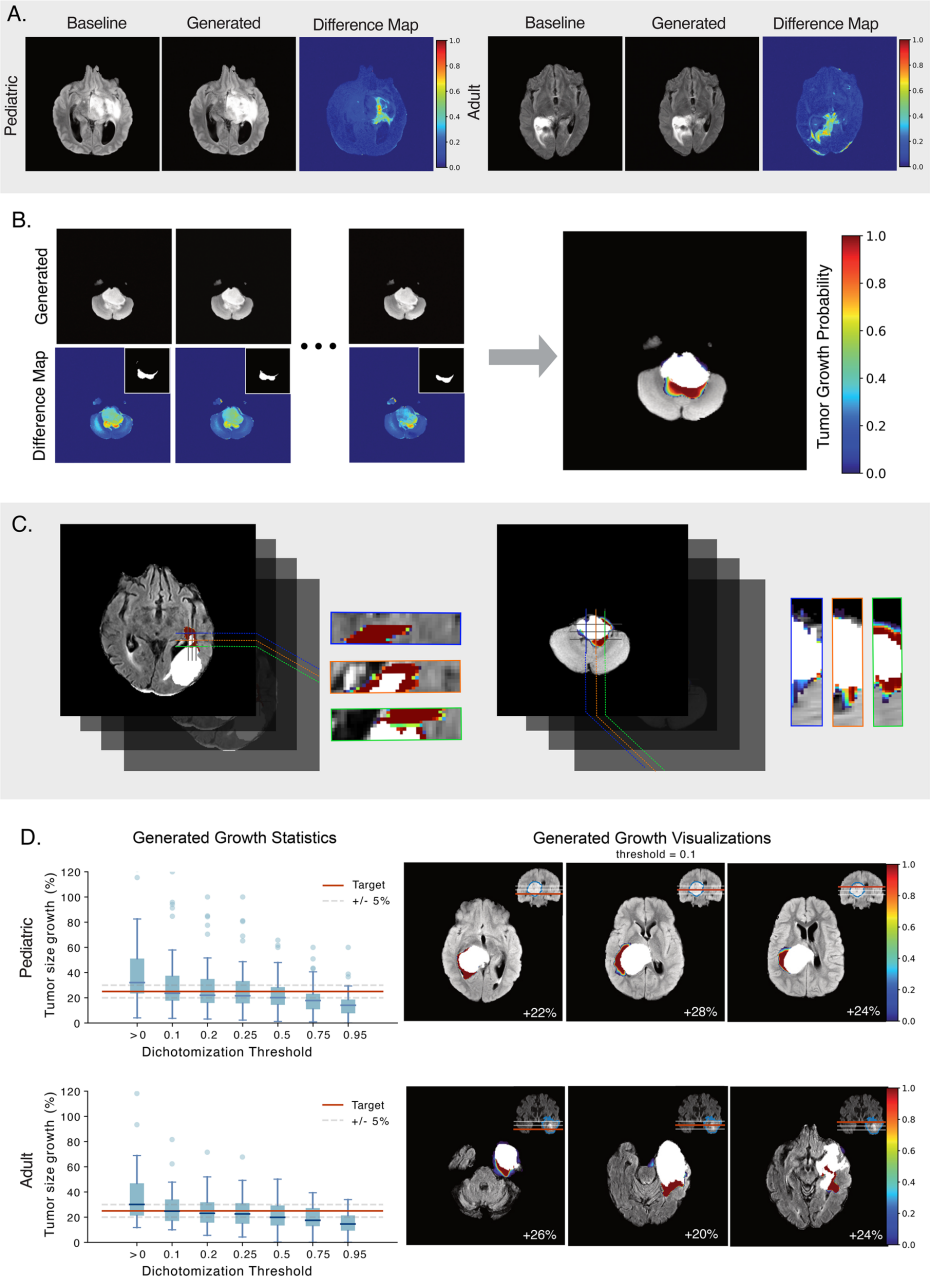
Relevant model components. **A** Representative examples of brain slices showing the generation of an enlarged tumor (+ 25%) from a pediatric (left) and an adult (right) patient. The difference maps show the pixel-wise changes of the generated image compared to the input (baseline). These highlight regions of change as a result of guided tumor growth. **B** Tumor growth probability map generation for one pediatric brain slice. Otsu thresholding is performed on the difference maps (bottom) to obtain tumor growth segmentations (upper right corner) which are aggregated into a growth map by averaging (right). **C** Stacking of axial view growth maps (*n* = 10) from a pediatric (left) and an adult (right) patient. **D** Quantification (left) and visualization (right) of tumor growth in the generated images. The images were guided towards a 25% tumor size increase. Quantification (left) shows the tumor growth distribution of generated images. Tumor size in the generated images is calculated at different dichotomization thresholds (> 0 to 0.95) over model tumor growth probability maps. Correlation plots between target and generated tumor areas calculated at a 0.1 dichotomization threshold are shown in Additional file 1: Figure S3. Visualizations (right) show representative pediatric and adult examples of growth maps at three different axial sections in the tumor region, shown together with their relative tumor growth at a 0.1 dichotomization threshold

Hyperparameter tuning (NL = 400, RS = 200k) results are summarized in (Additional file 1: Figure S2) as a trade-off between target-size achievement error and similarity metrics (PSNR, SSIM) and yielded a mean target error of 1.12 (4.5%). The optimized framework was applied to adult and pediatric brain tumor images to generate MRI scans in T2-FLAIR contrast displaying enlarged tumor areas of a predefined size. A 25% increase was chosen in line with RAPNO [30]. Representative difference maps obtained are displayed in Fig. 2A for an arbitrarily selected pediatric and adult patient. Notably, modifications of pixel values are confined to tumor neighboring regions while the characteristic anatomical features of the input brain slice are maintained. The observed tumor growth in the generated MRI scans aligns with expected physiological features.

To account for the stochasticity resulting from randomly sampling of noise in the forward process of the guided DDIM approach, the generative process can be repeated several times, starting from one input image. Figure 2B *(left)* highlights this probabilistic effect, showcasing generated images from different initializations. Difference maps from *n* = 100 inferences are compiled into a growth map (Fig. 2B, *right*). To investigate the 3D translation of our slice-based approach, independently generated consecutive axial slices’ growth maps were stacked (Fig. 2C), showcasing smooth transitions in coronal and sagittal planes.

The model’s performance was quantified through a comparison between observed tumor growth and the predefined target as percentage-wise increase (Fig. 2D). For a targeted 25% tumor growth, we report a mean increase of 24.1% for the adult samples and 23.6% for the pediatric for a probability threshold ≥ 0.1 removing pixels that were predicted as tumor in fewer than 10 of the 100 generations.

### Image quality assessment

**Fig. 3:**
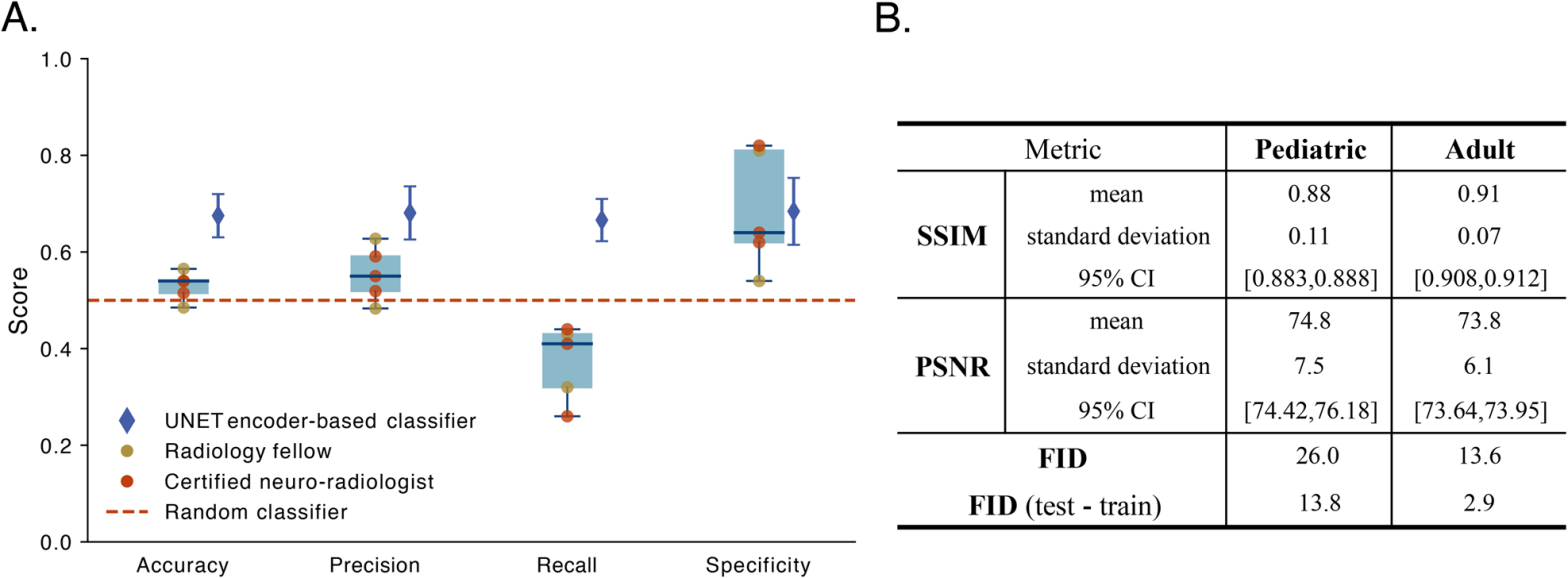
Image quality assessments. **A** Performance of human observers (colored points) in classifying real vs. generated images relative to a U-Net classifier (blue diamonds). The boxplots summarize the distribution of the metrics (accuracy, precision, recall, specificity) across the radiology experts (*n* = 5). **B** Summary of image quality metrics. For SSIM (structural similarity index measure) and PSNR (peak signal-to-noise ratio), we report mean, standard deviation and 95% confidence intervals (CI), computed based on Student’s t-distribution. One FID (Frechet inception distance) value is reported for each comparison as it represents distance between distributions. The FID between the real train and test set images serves as a baseline considering the dependency of the metric to dataset size. Metrics were only calculated for tumor containing slices in the test dataset (n_pediatric_ =1481, n_adult_=6164)

Human expert observers (*n* = 5) failed to distinguish real from generated MRIs in a blinded assessment, achieving an average accuracy of 0.53 (SD = 0.03) in the balanced dataset (Fig. 3A). While accuracy showed minimal variance across raters, specificity and recall distributions highlighted inter-rater variability. Notably, a greater proportion of samples was attributed to the generated category (65.7% on average).

Established metrics in the field of computer vision were applied to evaluate image quality of generated MRI (Fig. 3B). Lower FID values imply a narrower difference between the distribution of real and generated images. In our experiment, we observe a lower FID for the adult set both when considering absolute values (13.6 vs. 16.0) and when normalizing to baseline (distribution difference between train and test set, 10.7 vs. 12.2). However, the high SSIM and PSNR scores reveal a similar performance on average in preserving the characteristic structural components for the two datasets, with a higher variability in the case of pediatric samples.

**Fig. 4:**
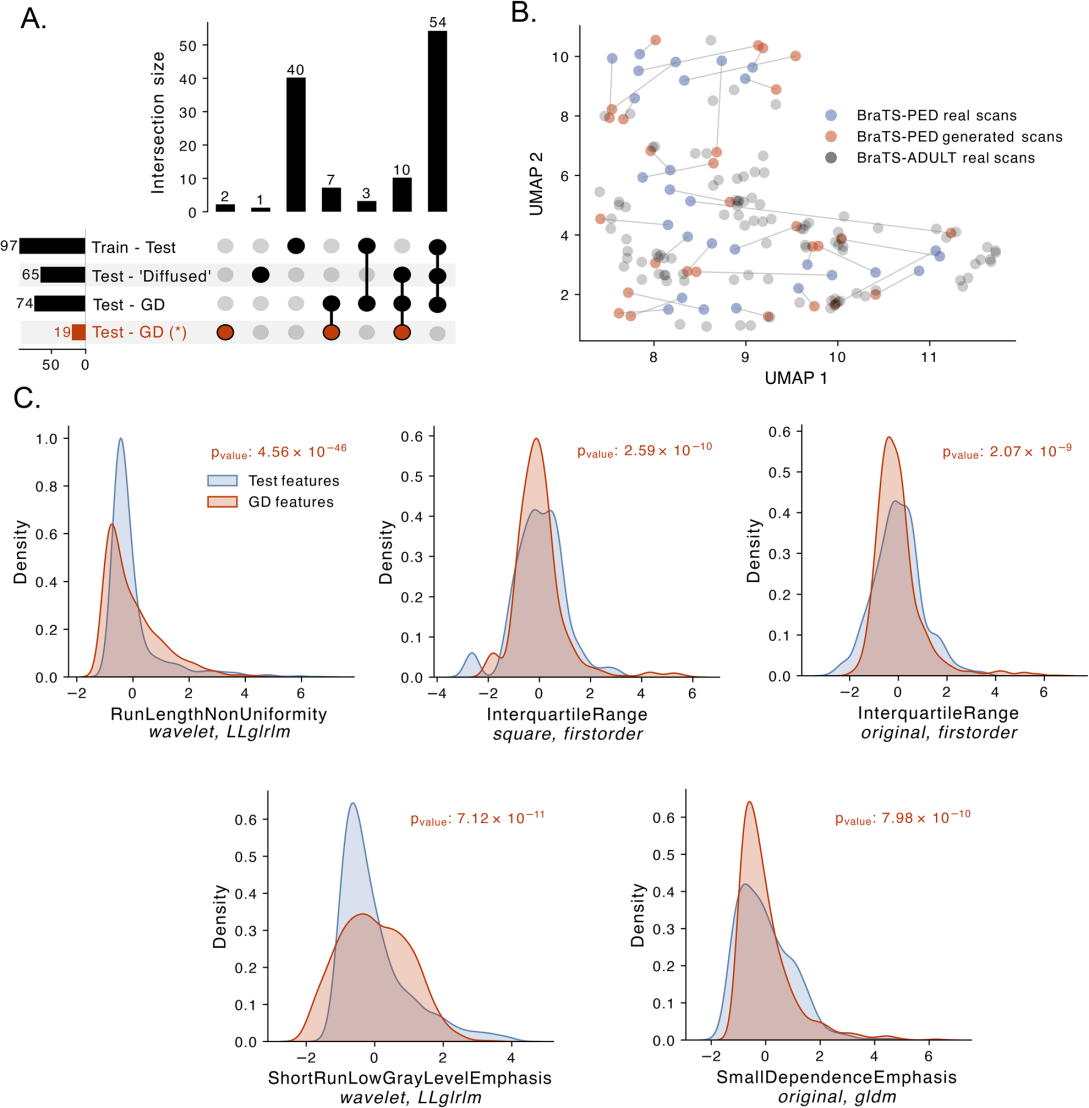
Radiomic features analysis. **A** Upset plot showing the different sets of radiomic features compared. The number of statistically significant features (alpha = 0.05, Bonferroni corrected) is shown on the horizontal histogram, while the overlap across these significant features is shown on the vertical bars. GD - guided diffusion with enlarged tumors; (*) - excluding significant features in ‘Train-Test’ comparison, ‘Diffused’ - reconstructed using the diffusion model, without tumor change guidance. **B** UMAP of pediatric real, adult real, and pediatric generated samples in the test sets. The U-MAP relies on radiomic features extracted from the axial central tumor slice for each patient. For the pediatric data, the paired samples are connected. **C** The distribution of most significant features in the ‘Test - GD (*)’ comparison. The adjusted p-value (Bonferroni corrected) is shown for each feature. The filter applied to the image and the class of features is specified on the x-axis

Figure 4A summarizes the radiomic features (RF) analysis. Statistical testing was initially conducted on the distributions of RF derived from full brain slices in the training and test sets, revealing a large proportion of significantly different features between these two subsets of real images. Subsequently, the test RFs were directly compared against ‘diffused’ images with no change in tumor size, identifying 32% of them as significantly different. However, 54 of the 65 identified features were present in the test-train comparison. Similarly, the comparison of RF for the generated images displaying enlarged tumors with their real counterpart showed that 57 out of the 74 significant features overlapped with those initially detected in the train-test analysis.

In a separate analysis, a reduced subset of features was tested. This was restricted to the non-significant subset from the analysis between real sets, revealing only 17% (19/111) of the features tested as significant. Taken together, these analyses highlight the high sensitivity of radiomic features across different subsets of real images and support the preservation of most RFs within the generative process. This is also supported by the shared distribution of the original and synthetic samples in the UMAP space (Fig. 4B).

Figure 4C shows the distributions of the five most significant radiomic features (lowest p-values) identified. Overall, we observed a smoother, more uniform distribution of the radiomic features derived from generated images. Three of them are centered around grey level aspects (RunLengthNonUniformity, ShortRunLowGrayLevelEmphasis, SmallDependenceEmphasis) with two of these representing high-order features extracted from the wavelet filtered images. The other two refer to the interquartile ranges, which inherently serve as a measure of the uniformity within the middle 50% of values. The observed patterns conform with expected results, taking into account the DDIM mode of operation. These types of generative models rely heavily on the denoising process which we see reflected in the most significant radiomic features which are directly related to lower levels of image noise. This results in a lack of ability to capture structured spurious artifacts in real MRI images during training and therefore less noisy generated scans.

### Longitudinal external evaluation

**Fig. 5:**
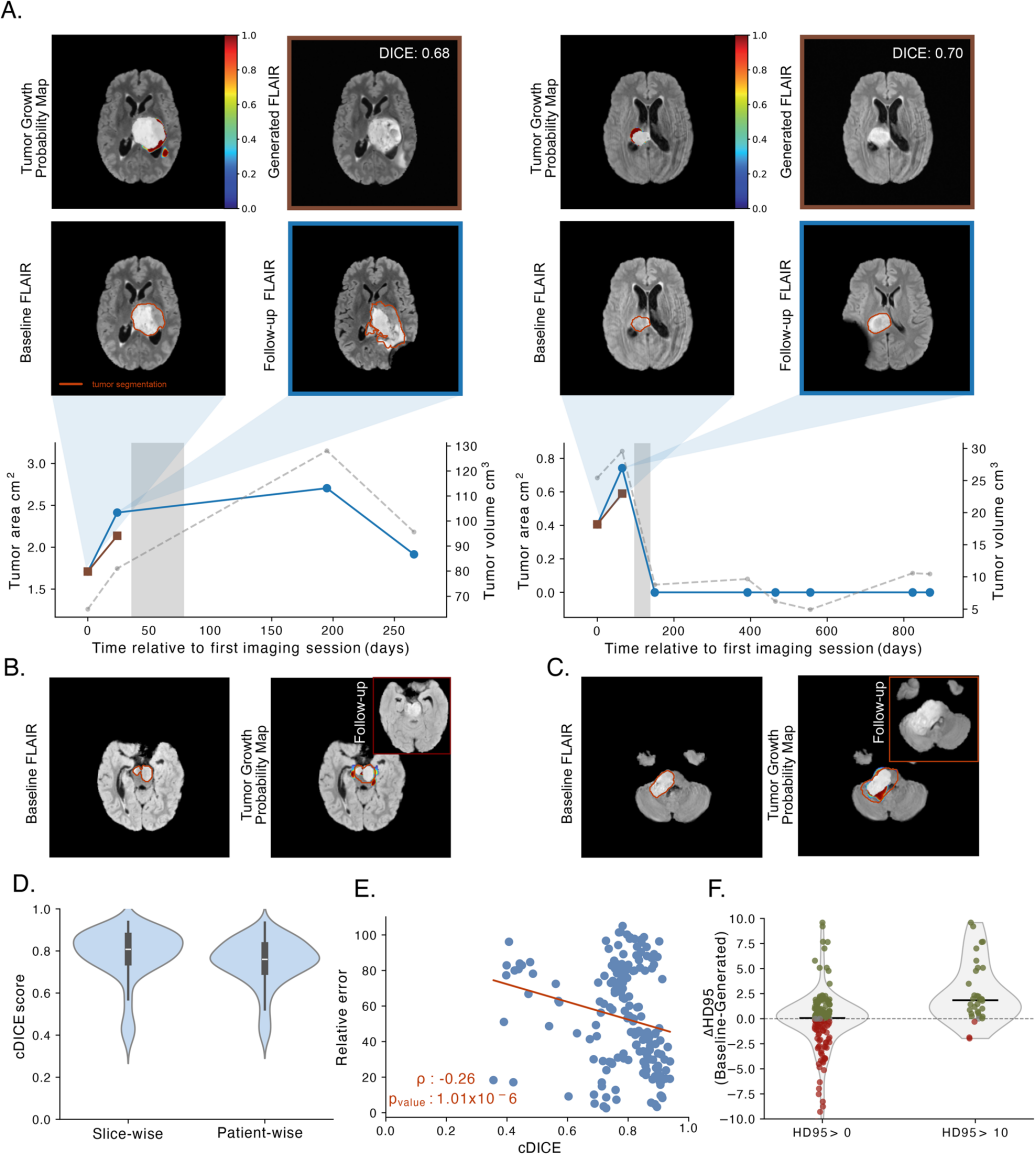
Longitudinal Evaluation. **A** Examples of longitudinal evaluation in pediatric DMG patients as T2-FLAIR contrast of the input, follow-up, and generated image with the predicted probability heatmap. The predictions are compared to the ground truth area (bottom blue) quantitatively: tumor area (bottom brown) and DICE of 0.68 and 0.70. The graph also shows radiotherapy delivery (grey shaded area) and the volume trajectory (cm^3^) over time (dashed grey line). Both patients show metal artifacts in the target image as a result of hearing aid implantation, part of symptom management. Note: the second example shows residual misalignment between baseline and follow-up scans due to imperfect coregistration. **B** Particularly poor example (DICE = 0.45) for the longitudinal validation. The growth map is shown overlaid to the baseline image together with the expected follow-up. **C** Good example (DICE = 0.81) for longitudinal validation. The growth map is shown overlaid to the baseline image together with the expected follow-up. **D** Violin plot of the continuous DICE distribution for the longitudinal evaluation aggregated at the slice-level (*n* = 178) as well as at patient level (*n* = 13). **E** Correlation plot and Spearman ρ between cDICE scores and error of the regressor predicting relative tumor size. **F** Violin plots showing the change in 95th percentile Hausdorff distance (ΔHD95) between baseline-to-follow-up and generated-to-follow-up comparisons, stratified by cases with HD95 > 0 and HD95 > 10 (growth observed furthest away from baseline). Individual data points are overlaid, with green indicating accurate directionality prediction and red indicating poor prediction. Horizontal black lines denote the median

We generated longitudinal follow-up images in a pre-treatment setting as tumor growth probability maps in the external cohort. Qualitative examples illustrate the predicted tumor growth probability map for representative cases, including a challenging case characterized by poor temporal coregistration (Fig. 5A). Additional examples highlight a particularly poor (Fig. 5B) and good (Fig. 5C) example. We find excellent alignment between the predicted and true directionality of tumor growth and a good spatial agreement of the whole tumor area (mean cDICE score of 0.79 at the slice-level (SD = 0.13, 95% CI=[0.767, 0.805] based on Student’s t-distribution), Fig. 5D), emphasizing the generalizability of our approach to data from other institutions. Full metrics at the patient and scan-level are provided in Additional file 1: Table S3 and Table S4.

We further observe a significant negative correlation (Spearman’s ρ: -0.26, p_value_=1 × 10^− 6^) of modest effect size between the error in tumor size estimation and the cDICE score of the predicted spatial tumor growth (Fig. 5E). This supports our hypothesis that the inaccurate tumor size prediction of the regressor propagates to the guided diffusion process, flagging a failure of the generative framework in such cases. In contrast, no significant correlation was observed between the specific growth rate and cDICE (Spearman’s ρ: -0.12,  p-value= 0.11), suggesting that tumor growth itself does not systematically drive model performance (Additional file 1: Figure S4). Thus, regressor performance on baseline relative tumor size from input MRIs serves as a practical proxy for assessing the reliability of the overall prediction. Furthermore, the generated follow-up images demonstrated lower HD95 distances to the target compared to the baseline images (Fig. 5F, mean ∆HD95 = 0.1, SD = 2.5), confirming the directional tumor growth. While overall improvement is small, a substantial improvement is particularly seen for samples where the baseline is furthest from the follow-up (HD95 > 10, mean ∆HD95 = 2.5, SD = 2.9).

## Discussion

We present a proof-of-principle to harness generative AI models for conditional modeling of spatial tumor growth patterns, with specific application to pediatric DMG imaging. This approach has strong potential to inform clinical applications, such as radiotherapy treatment volume definition according to likely tumor growth trajectories. Our approach yields promising results, demonstrating the ability to abstract complex patterns of tumor progression across related disease domains, here GBM and pediatric high-grade glioma to infer DMG growth in the light of limited pediatric data.

While the applications of conditional DDPMs have predominantly focused on synthetic image generation or modality translation, recent studies demonstrate the potential of guided diffusion approaches in constraining generations to specific anatomical boundaries [17, 31–34]. Notably, the diffusion models learn these constraints during training, and do not require explicit anatomical inputs such as landmarks or atlases. Building on the work of Wolleb et al., we extend the framework and apply it to a generative predictive task, enabling longitudinal spatial growth predictions. Focusing on the pathology of DMGs enabled comparisons to true tumor evolution in a way that is rarely available. Our model shares conceptual similarities with the work of Liu et al. [7], leveraging a conditional diffusion model for anatomically guided tumor progression. However, while Ta-Diff incorporates treatment conditioning, our model uses a regressor-guided DDIM to enforce relative tumor size control in a pediatric cohort without requiring longitudinal training data. This enables applications in data-scarce settings, though further extensions could also incorporate treatment effects.

The value of our validation relies on the external DMG dataset acquired through a multi-institutional international collaboration. Importantly, we generate tumor growth probability maps and validate our predictions through quantitative comparisons against real longitudinal brain MRI images, achieving a mean continuous DICE of 0.79. While commonly applied for evaluating segmentation performance, here we employ the continuous DICE score primarily as a proxy for spatial agreement between predicted probabilities and observed tumor regions. Given the sensitivity of DICE to misalignments caused by imperfect longitudinal coregistration of baseline and follow-up scans, we interpret these scores cautiously and emphasize the directional fidelity of the predicted spatial growth, as shown by mean ∆HD95 of 2.5 in samples where growth is observed furthest away from the tumor at baseline (HD95 > 10).

The integration of such predictions in the clinical landscape of DMGs holds significant potential to enhance the standard of care. Availability of tumor growth probability maps highlighting the most likely direction of progression, as presented in this work, could revolutionize RT treatment planning. Indeed, target volume definitions are a key factor increasingly recognized for its extensive impact on therapy outcome [35]. Currently, this relies on guidelines recommending fixed-size isotropic margins and the clinician’s experience [36]. Notably, for this application, accurately capturing the direction of growth holds greater significance than the precise replication of anatomical and physiological features present in the target image. We emphasize the correlation between the regressor error and spatial prediction performance (DICE), which could serve as a proxy for assessing prediction reliability. Despite this insight, the degree of deviation that would clinically still be acceptable for RT target definition warrants further validation and may depend on the individual case, specifically on the anisotropic extent of margin adaptation proposed. Additionally, upon further validation, such predictions could serve as the basis for a synthetic control arm in prospective clinical trials, addressing a critical bottleneck in the discovery of novel therapies for DMGs through simulation of “what a tumor would have looked like in the absence of treatment”. While here we showcase the direct implications for DMG tumors, the application of this framework could be extended to model tumor growth in other body parts or to address similar longitudinal predictive tasks.

Another key contribution presented in this work is the comprehensive evaluation of image quality in the context of different application horizons. We leverage both established quantitative measures for computer vision, expert ratings and radiomic features. These diverse qualitative and quantitative assessments capture different aspects of realistic MRI generation, emphasizing the particularities of medical image synthesis. We demonstrate state-of-the-art SSIM scores as well as a low FID value for the adult subset, compared to the real images. Interestingly, while the pediatric subset shows a higher FID score, trained radiologists are unable to distinguish between the generated and real images, a trend previously mentioned in literature [37]. These observations reinforce and align well with previous literature highlighting the limitations of such metrics developed on and for large natural images datasets [26, 38]. Although the content of the generated MRI scans appears realistic and indistinguishable from that of real images to expert raters, subtle differences in statistics derived from pixel values render generated and real images significantly different for machine-readable applications (mean accuracy of CNN classifier of 0.69 on a balanced dataset). These findings underline the importance of considering the application context of synthetic images and raise concerns regarding the potential impact of such differences on downstream models relying on hybrid or purely synthetic data [39, 40].

While this study presents important advancements toward the use of generative models as predictive tools for anatomical DMG growth, it is essential to acknowledge some limitations. Firstly, due to the computationally intensive inference procedures and limited data, we restrict our research to axial 2D slices. We account for this limitation by analyzing stacking of axial plane predictions and supporting qualitative examples. Secondly, while a strength of our approach is training on single-timepoint data, an assumption of the follow-up tumor size is warranted for inference. This limits applications for end-to-end longitudinal prediction in the current format but could be mitigated through a mechanistic learning approach, integrating the modeling of target tumor volume with the generative DDIM framework based on longitudinal training data. Lastly, due to the strict requirement for consecutive pre-treatment imaging sessions, the number of longitudinal evaluation samples was limited. However, while restricted to DMG patients, our dataset spans multiple institutions, introducing substantial variability in acquisition protocols, imaging hardware and patient demographics. Future research should focus on extending the predictive framework to 3D space, removing the requirement of specifying the target tumor size pre-hoc. For the creation of a fully predictive framework, one could explore a similar architecture using time between imaging sessions as an additional conditioning term or adopt video-generative models with an imaging time series input to fully abstract growth rate. Additionally, using a separate approach for modelling tumor burden would enable feeding that prediction directly in the current architecture as we show in [41]. While very elegant, this solution limits the applicability of the model towards the later time in the patient trajectory. Further work should also consider the inclusion of additional patient-specific clinical values (age, treatment course, etc.) towards the generation of a more individualized prediction. Notably, incorporating the baseline tumor segmentation as an input conditioning term, not used here, could improve tumor localization and spatial accuracy. Continued validation and refinement of these models will be critical for their translation to clinical practice, particularly across other tumor types and disease contexts.

## Conclusions

Our study provides a meaningful step forward in the application of generative AI models for predicting anatomically constrained tumor growth on clinical brain MRI, producing high-quality predictions that accurately conform to growth patterns in clinical cases. The promising results highlight the potential of DDIMs to abstract complex tumor progression patterns and generate clinically relevant tumor growth probability maps, while highlighting the need to understand the content of synthetic data. These advancements could enable more precise radiotherapy target volume definition and address critical bottlenecks in novel therapy discovery.

## Supplementary Information

Below is the link to the electronic supplementary material.

Supplementary Material 1: Figure S1–Figure S4. Table S1–Table S4.

Supplementary Material 2

## Data Availability

Training data is publicly available at the Synapse Repository (https://synapse.org/Synapse: syn51156910) upon request. This includes the BraTS23 Adult Glioma dataset [20] and the BraTS23 Pediatric dataset [21].The imaging longitudinal external dataset is available upon request with a relevant data usage agreement (DUA) from the data providing institution. Access is restricted due to patient privacy and ethical/legal constraints governing clinical imaging data. Requests should be directed to Prof. Sabine Mueller, DMG Center Zurich, University Children’s Hospital Zurich and University of California San Francisco (sabine.mueller@ucsf.edu). Requests will be reviewed on a case-by-case basis; a response timeframe and DUA conditions will be communicated upon initial contact. Data use is subject to restrictions on downstream reuse and may include authorship requirements as specified in the DUA. Our code is available at https://gitlab.ethz.ch/BMDSlab/publications/oncology/guided-denoising-diffusion-models-for-brain-tumor-mri.

## Abbreviations

DMG: diffuse midline glioma
GBM: glioblastoma
MRI: magnetic resonance imaging
DDPM: denoising diffusion probabilistic model
DDIM: denoising diffusion implicit model
FID: Frechet inception distance
SSIM: structural similarity index measure
PSNR: peak signal to noise ratio
RF: radiomic features
NL: noise level
RS: regressor scale
FLAIR: fluid-attenuated inversion recovery
