# Supplementary material for: Generative AI for spatial tumor growth on MRI: a proof-of-principle study in pediatric diffuse midline glioma
Source: BMC Med. 2026 May 18;24:389. doi: 10.1186/s12916-026-04911-y (PMC13352721; doi:10.1186/s12916-026-04911-y)
Supplement: Supplementary file 1 — Supplementary Material 1: Figure S1–Figure S4. Table S1–Table S4. [file 12916_2026_4911_MOESM1_ESM.docx]

Additional file 1


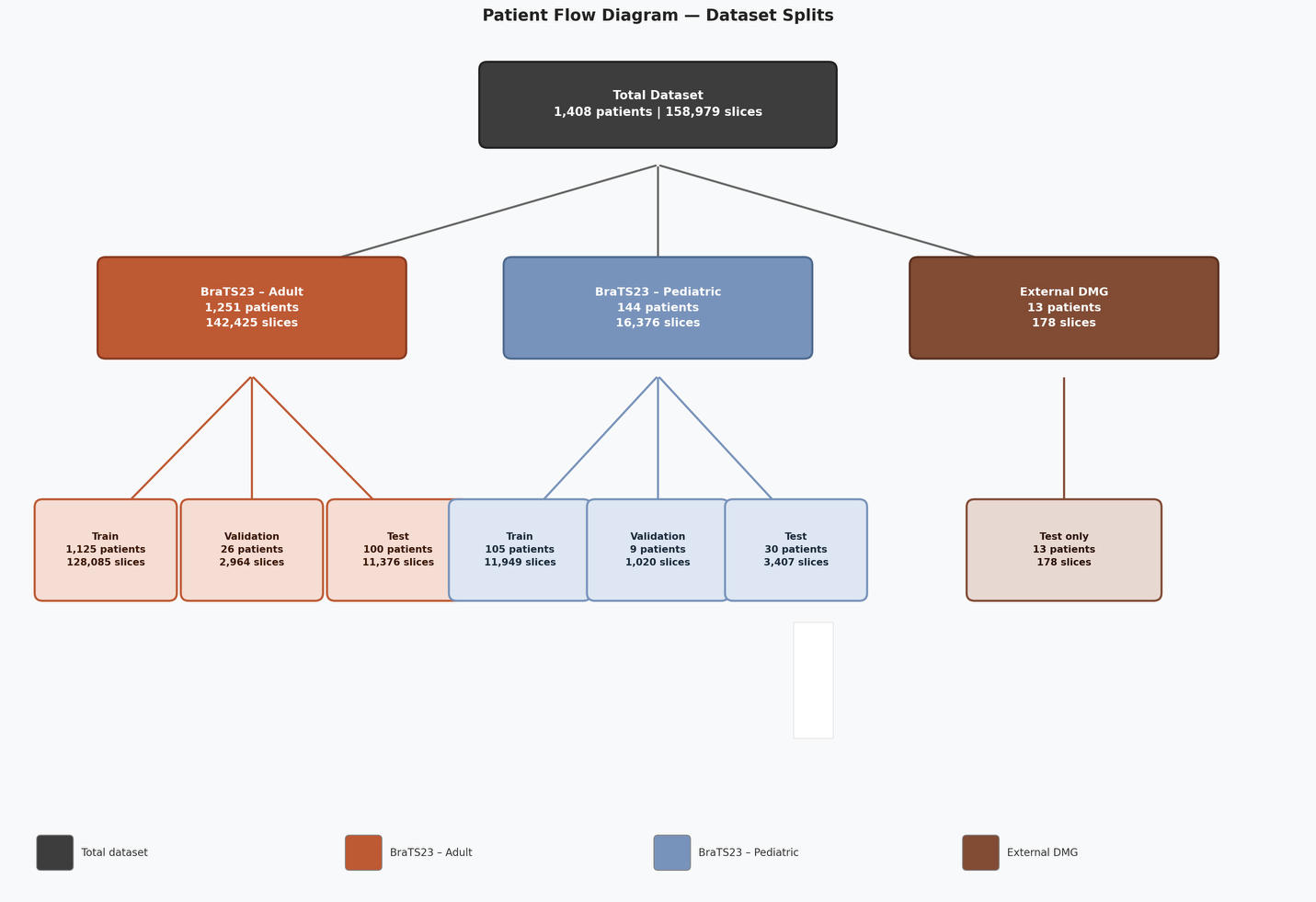


*Figure S1.* **Overview of datasets and data partitioning.** Hierarchical breakdown of the three datasets included in this study: BraTS23 – Adult (n = 1,251), BraTS23 – Pediatric (n = 144), and an external diffuse midline glioma (DMG) cohort (n = 13). BraTS23 datasets were partitioned into training, validation, and test sets, while the external DMG cohort was used exclusively for testing.

*Figure S2.* **Hyperparameter tuning**. All values are calculated for the tumor containing slices of the BraTS23-PED validation dataset. (A) Bland-Altman plot showing the error calculated as the difference between the set target size and the one observed in the generated images. The mean and 95% range are displayed for different noise levels (NL=300, NL=400, NL=500), while the regressor scale (RS) is set to 200k. (B) Boxplot showing peak signal to noise ratio (PSNR) and structural similarity index measure (SSIM) for different noise levels, while RS=200k. (C) Bland-Altman plot showing the error calculated as the difference between the set target size and the one observed in the generated images. The mean and 95% range are displayed for different regressor scales (RS=100k, RS=200k, RS=500k), while the NL=400. (D) Boxplot showing PSNR and SSIM for different regressor scales, while NL=400.

*Figure S3.* **Spearman correlation between target and generated tumor areas.** Scatter plots show the correlation between the target and generated area for total tumor area and growth area in the (A) pediatric and (B) adult test datasets. In both cohorts, total tumor area showed near-perfect correlation (Spearman r = 0.99, p < 0.05). Growth area correlation was moderate in the pediatric dataset (Spearman r = 0.71, p < 0.05) and fair in the adult dataset (Spearman r = 0.51, p < 0.05). Areas are calculated by using a dichotomization threshold of 0.1 (see Methods section).

*Figure S4.* **Relationship between specific growth rate (SGR) and cDICE performance for the longitudinal DMG dataset**. Scatter plot showing the Spearman correlation between SGR and cDICE computed on the whole tumor across all test cases. SGR was computed as ln(V₂/V₁)/Δt, where V₁ and V₂ denote tumor volumes at the baseline and follow-up timepoints respectively, and Δt is the interscan interval in days.

**Supplementary Tables**

*Table S1*. **Train, validation and test splits for the BraTS-ADULT, BraTS-PED and External DMG datasets.** All splits were done at the patient level. No demographic information was made available to the authors of the study for either dataset.

|  | **Train** | | **Validation** | | **Test** | | **Total** | |
| --- | --- | --- | --- | --- | --- | --- | --- | --- |
|  | Patients | Slices | Patients | Slices | Patients | Slices | Patients | Slices |
| **BraTS23 - ADULT** | 1125 | 128,085 | 26 | 2,964 | 100 | 11,376 | 1251 | 142,425 |
| **BraTS23 - PED** | 105 | 11,949 | 9 | 1,020 | 30 | 3,407 | 144 | 16,376 |
| **External DMG** | - | - | - | - | 13 | 178 | 13 | 178 |

Table S2. **Summary statistics for the longitudinal external dataset.** Slice-wise metrics for the selected slices (n = 178) are reported as mean, standard deviation (SD), and Student's t-distribution-based 95% confidence intervals (CI). Metrics include the baseline tumor area (cm^2^), follow-up tumor area (cm^2^), and percentage of tumor area increase. The same quantification is provided for the full 3D test image pairs (n = 23) drawn from the 13 patients in the longitudinal evaluation dataset in cm^3^. Note that all 23 pairs correspond to images acquired prior to radiotherapy.

|  | **Metric** | **Mean** | **SD** | **Lower CI** | **Upper CI** | **n** |
| --- | --- | --- | --- | --- | --- | --- |
| Slice-wise  (2D) | Baseline areas  (cm^2^) | 5.59 | 3.98 | 5 | 6.18 | 178 |
|  | Follow-up areas  (cm^2^) | 7.46 | 5.19 | 6.7 | 8.23 | 178 |
|  | Percentage increase  (%) | 37.57 | 23.96 | 34.02 | 41.11 | 178 |
| Volume-wise  (3D) | Baseline volumes  (cm^3^) | 30.88 | 24.07 | 20.47 | 41.29 | 23 |
|  | Follow-up volumes  (cm^3^) | 31.14 | 19.87 | 22.55 | 39.73 | 23 |

*Table S3.* **Patient-level performance on the longitudinal external dataset**. DICE and continuous DICE (cDICE) are reported per patient (n = 13) for two regions of interest: the full tumor area and the growth region (defined as the area of tumor area increase between baseline and follow-up). Metrics are computed slice-wise across all 178 selected MRI slice pairs and averaged per patient.

| **PID** | **DICE (full)** | **cDICE (full)** | **DICE (growth)** | **cDICE (growth)** |
| --- | --- | --- | --- | --- |
| pid_1 | 0.766 | 0.824 | 0.249 | 0.171 |
| pid_2 | 0.680 | 0.814 | 0.239 | 0.313 |
| pid_3 | 0.789 | 0.857 | 0.397 | 0.241 |
| pid_4 | 0.718 | 0.851 | 0.269 | 0.175 |
| pid_5 | 0.741 | 0.759 | 0.254 | 0.081 |
| pid_6 | 0.744 | 0.809 | 0.322 | 0.250 |
| pid_7 | 0.453 | 0.439 | 0.190 | 0.071 |
| pid_8 | 0.551 | 0.526 | 0.370 | 0.24 |
| pid_9 | 0.817 | 0.827 | 0.387 | 0.246 |
| pid_10 | 0.743 | 0.777 | 0.271 | 0.200 |
| pid_11 | 0.868 | 0.877 | 0.410 | 0.376 |
| pid_12 | 0.672 | 0.651 | 0.331 | 0.222 |
| pid_13 | 0.839 | 0.835 | 0.271 | 0.121 |

*Table S4.* **Scan-level performance on the longitudinal external dataset.** DICE and continuous DICE (cDICE) are reported per session (SES) (n = 23) for two regions of interest: the full tumor area and the growth region (defined as the area of tumor area increase between baseline and follow-up). Metrics are computed slice-wise across all 178 selected MRI slice pairs and averaged per patient and per session.

| **PID** | **SES** | **DICE (full)** | **cDICE (full)** | **DICE (growth)** | **cDICE (growth)** |
| --- | --- | --- | --- | --- | --- |
| pid_1 | ses_1 | 0.743 | 0.777 | 0.271 | 0.200 |
| pid_2 | ses_1 | 0.810 | 0.889 | 0.290 | 0.271 |
|  | ses_2 | 0.710 | 0.716 | 0.312 | 0.159 |
|  | ses_3 | 0.791 | 0.870 | 0.205 | 0.160 |
|  | ses_4 | 0.798 | 0.848 | 0.258 | 0.160 |
|  | ses_5 | 0.721 | 0.797 | 0.182 | 0.105 |
| pid_3 | ses_1 | 0.737 | 0.810 | 0.170 | 0.108 |
|  | ses_2 | 0.748 | 0.809 | 0.414 | 0.336 |
| pid_4 | ses_1 | 0.828 | 0.793 | 0.491 | 0.251 |
|  | ses_2 | 0.828 | 0.854 | 0.223 | 0.176 |
|  | ses_3 | 0.794 | 0.834 | 0.445 | 0.311 |
| pid_5 | ses_1 | 0.672 | 0.651 | 0.331 | 0.222 |
| pid_6 | ses_1 | 0.764 | 0.860 | 0.334 | 0.230 |
|  | ses_2 | 0.677 | 0.844 | 0.212 | 0.127 |
| pid_7 | ses_1 | 0.453 | 0.439 | 0.190 | 0.071 |
| pid_8 | ses_1 | 0.551 | 0.526 | 0.370 | 0.240 |
| pid_9 | ses_1 | 0.839 | 0.835 | 0.271 | 0.121 |
| pid_10 | ses_1 | 0.789 | 0.857 | 0.397 | 0.241 |
| pid_11 | ses_1 | 0.868 | 0.877 | 0.410 | 0.376 |
| pid_12 | ses_1 | 0.741 | 0.759 | 0.254 | 0.081 |
| pid_13 | ses_1 | 0.680 | 0.814 | 0.239 | 0.313 |
