# Supplementary material for: Generative AI for spatial tumor growth on MRI: a proof-of-principle study in pediatric diffuse midline glioma
Source: BMC Med. 2026 May 18;24:389. doi: 10.1186/s12916-026-04911-y (PMC13352721; doi:10.1186/s12916-026-04911-y)
Supplement: Supplementary file 2 — Supplementary Material 2 [file 12916_2026_4911_MOESM2_ESM.docx]

Additional file 2

## **Model Architecture and Training**

In the first stage, a denoising diffusion probabilistic model (DDPM) [[11]](https://paperpile.com/c/YnlAYH/vPYkP) and a regression model predicting tumor size are trained separately. The DDPM (113,681,160 parameters) used a learning rate of 10^-4^ and one attention head set to a resolution of 16. The model was trained for 160,000 steps using the Adam optimizer and following the hybrid loss described by Nichol and Dhariwal.[[22]](https://paperpile.com/c/YnlAYH/lC3UP)

The regression model (5,452,833 parameters) was trained using a learning rate of 10^-4^, attention heads at resolution 8,16 and 32 and setting the depth of the model to 4. The mean squared error (MSE) loss function, and the Adam optimizer were used during the training process (50,000 epochs). The models were jointly trained on the adult and pediatric cohorts.

During the generative phase, first, a noised version of the input image (x(L), for L diffusion steps) is obtained using a denoising diffusion implicit model (DDIM) formulation [[10]](https://paperpile.com/c/YnlAYH/NcyQk). This is done following the DDIM forward diffusion process, while setting the weights as given by the trained DDPM. Once x(L) is known, the sampling is carried out. At each step, from L to 0, the noise is gradually removed as determined by the learned diffusion parameters in a structured fashion guided by the gradient of the regressor. For this the target relative tumor size desired in the output image is set. Apart from the constant weight defining the influence of the gradient (regressor scale RS and constant c), another scaling factor is added to the gradient term (r_t_) which gets updated at every step t (Eq.1). This depends on the target tumor size and the predicted one and works in a feedback-like manner, increasing the influence when the current size is far from the desired one and adjusting the sign of the gradient to grow or shrink the tumor. RT, c and r_t_ together define the magnitude of the gradient at every step of the denoising (Eq. 2, Eq. 3, where $\bar{\epsilon_{\theta}}$ combines the gradient guidance term and the diffusion model predicted noise $\epsilon_{\theta}$ ). The noise level (NL) of the generative process determines noise addition to the input image and consequently the number of inference steps, impacting the preservation of the sample’s original anatomical features. Each sampling process for generating one slice with an enlarged tumor takes 113s. In this work, the optimal RS and NL are selected based on overall image quality metrics (SSIM, PSNR) and relevant measures assessing tumor growth: error to target.

$r_{t}\leftarrow i-R\left( x_{t},t \right)$ (Eq. 1)

$\bar{\epsilon_{\theta}}\leftarrow\epsilon_{\theta}\left( x_{t},t \right)-RS r_{t} c \nabla_{x_{t}}R\left( x_{t},t \right)$ (Eq. 2)

$x_{t-1}\leftarrow f\left( x_{t},\bar{\epsilon_{\theta}} \right)$ (Eq. 3)

1. **Hyperparameter Tuning**

The achievement of the target size during inference is explored for different parameter combinations on the BraTS test set through error and image similarity metrics as shown in **Figure S2**. Panel A shows diminishing mean target achievement errors with increasing noise levels, matching the anticipated DDIM behavior. Increasing the number of noising steps provides more flexibility for the model during the reverse diffusion process. These findings were considered in combination with the decreasing PSNR and SSIM values for the tumor area as shown in Figure S1B as a way of balancing target achievement and maintaining the characteristic features of the initial tumor. Similarly, increasing RS values reduces the observed error (Figure S2C), demonstrating a drop in the average from 1.36 for RS=100k to 0.69 for RS=500k. However, this error reduction comes at the cost of a marked decrease in similarity metrics. Taken together with visual assessment of resulting generated images, RS=200k was chosen as the optimal parameter for further analyses. While NL=500 shows medians closest to 0 for error, we observe a widening 95% range of values when increasing NL from 400. Compared to NL=300, using 400 steps maintains the 95% range while decreasing error. This coupled with the decreasing SSIM as NL increases, led to choosing NL=400 as optimal.

Notably, both panels S2A and S2C highlight the positive correlation between the mean target relative tumor size and the observed error. Furthermore, samples with smaller tumor areas tend to form a defined cluster which is particularly affected by parameter variations, typically showing greater error values with increasing NL or RS.

1. **U-Net segmentation model**

For tumor segmentation, a standard U-Net architecture is developed internally, relying on the original implementation^23^, using the T2-FLAIR modality as single-channel input. The network is composed of four encoder blocks and four decoder blocks, connected by a bridge. Each encoder block contains two 3x3 convolution, followed by a ReLu (rectified linear unit) activation function and a 2x2 max-pooling layers. The final layer reduces the dimensions of the features by half. Each decoder block contains a 2x2 transpose convolution which doubles the spatial dimensions followed by two 3x3 convolution and ReLu. The final decoder block contains a 1x1 convolution with a sigmoid activation function. The result of the final layer is a classification of each pixel either as a tumor or a healthy pixel.

Given the different features of pediatric and adult gliomas, the networks are trained separately for the two tasks. For the adult tumor segmentation, the model is trained on the adult BraTS23-ADULT training subset for 100,000 steps. The average Sorensen-Dice Coefficient (DICE) obtained on the validation subset for the slices containing tumor represents the stopping criteria. For the pediatric tumor segmentation, the model is initially trained on the adult BraTS23-ADULT training subset as described above. Then, the weights of the first two encoder block are frozen and the network is trained for a further 3,000 steps on the pediatric BraTS23-PED subset. The same stopping criteria as for the adult tumor segmentation is applied here.

1. **U-Net Classifier**

The classifier follows the architecture of the encoder of the diffusion model's U-Net-like network, excluding the time-step embeddings. The model takes as input an image and the final encoder layer, followed by a sigmoid activation function, predicts a label: healthy or diseased. The number of channels in the first layer is set to 32 and the depth of the classifier is set to 4. The model uses attention heads at resolution of 8, 16 and 32. The model is trained on the joint BraTS23-GLI and BraTS23-PED real dataset and corresponding generated images (with either 25% larger tumors or 25% smaller tumors), using the Binary Cross Entropy Loss. Only tumor-containing slices are used, and the positive prevalence of the training dataset is 50%. Training is done with a learning rate of 10^-4^ using the Adam optimizer.
